# Possible Roles of Ectophosphatases in Host-Parasite Interactions

**DOI:** 10.1155/2011/479146

**Published:** 2011-04-26

**Authors:** Marta T. Gomes, Angela H. Lopes, José Roberto Meyer-Fernandes

**Affiliations:** ^1^Instituto de Bioquímica Médica, Universidade Federal do Rio de Janeiro, CCS, Bloco H, Cidade Universitária, Ilha do Fundão, 21941-902 Rio de Janeiro, RJ, Brazil; ^2^Instituto Nacional de Ciência e Tecnologia de Biologia Estrutural e Bioimagem (INCTBEB), CCS, Bloco H, Cidade Universitária, 21941-902 Rio de Janeiro, RJ, Brazil; ^3^Instituto de Microbiologia Professor Paulo de Góes, Universidade Federal do Rio de Janeiro, CCS, Bloco H, Cidade Universitária, Ilha do Fundão, 21941-902 Rio de Janeiro, RJ, Brazil

## Abstract

The interaction and survival of pathogens in hostile environments and in confrontation with host immune responses are important mechanisms for the establishment of infection. Ectophosphatases are enzymes localized at the plasma membrane of cells, and their active sites face the external medium rather than the cytoplasm. Once activated, these enzymes are able to hydrolyze phosphorylated substrates in the extracellular milieu. Several studies demonstrated the presence of surface-located ecto-phosphatases in a vast number of pathogenic organisms, including bacteria, protozoa, and fungi. Little is known about the role of ecto-phosphatases in host-pathogen interactions. The present paper provides an overview of recent findings related to the virulence induced by these surface molecules in protozoa and fungi.

## 1. Introduction

Cells are exposed to diverse environmental stimuli throughout their cycles in all biological systems. Protein phosphorylation and dephosphorylation are central events in cell recognition of external and internal signals, leading to specific responses. While protein kinases transfer a phosphate group from ATP to a protein (i.e., phosphorylate), protein phosphatases catalyze the removal of phosphate groups from specific residues of proteins (i.e., dephosphorylate) [1, 2]. The balance between the antagonistic activities of protein kinases and phosphatases are responsible for many cellular functions, including metabolic pathways, cell-cell communication, proliferation, and gene transcription [3]. 

 The complete genome sequencing of various microorganisms made it possible to assemble the kinome and phosphatome of a few trypanosomatids [4, 5]. These strategies have brought new perspectives of researches in the areas of biochemistry, physiology, and genetics, providing knowledge about the microorganisms' life cycles, as well as predicting diagnostic biomarkers, novel drug targets and vaccine candidates against parasitic infections. 

Parasites engage a plethora of surface and secreted molecules in order to attach and enter mammalian cells. Some of these molecules are involved in triggering specific signaling pathways both in the parasite and the host cell, which are critical for parasite entry and survival [6]. Plasma membranes of cells contain enzymes that are oriented with their active sites facing the external medium rather than the cytoplasm, which are important for host-parasite interactions [7, 8]. In the case of an ectoenzyme other criteria can be included as: (1) the enzyme has to act on extracellular substrate, (2) cellular integrity is maintained during enzyme activity, (3) the products are released extracellularly, (4) the enzyme is not released to the extracellular environment; and (5) the enzyme activity can be modified by nonpenetrating reagents [7, 8]. Supporting this idea, the presence of surface-located phosphatases, called ecto or extracytoplasmic phosphatases have been characterized in several microorganisms. However, the physiological roles of these enzymes in these cells are not well established yet. In eukaryotes, the most predominant phosphorylation sites are detected on serine, threonine and tyrosine residues. Thus, catalytic signature motifs and substrate preferences classified these proteins into four major groups: phosphoprotein phosphatases (PPPs), metallo-dependent protein phosphatases (PPMs), aspartate-based phosphatases with a DxDxT/V motif (the members of these three groups are Ser/Thr specific phosphatases) and the distinct group of protein tyrosine phosphatases (PTPs) [9]. Protein tyrosine phosphatases belong to three evolutionarily unrelated classes: protein tyrosine phosphatases (PTPs), Cdc25 and low molecular weight phosphatases (LMW-PTPs), which have a common motif (CX5R) in their catalytic sites [10]. The classical PTPs are classified, depending on the presence or absence of transmembrane domains, into receptor or nonreceptor type phosphatase groups. 

The use of inhibitors, divalent cations, metal chelators and different pH range has also been an important tool for classification of these enzymes. Likewise, phosphatases may be acid or alkaline according to their pH range for activity. The optimum pH for acid ectophosphatases lies on the acid range (pH values between 4.5 and 5.5), while the optimum pH for alkaline ectophosphatases lies on the alkaline range (pH values between 8.0 and 9.0) [9, 10]. The inhibitors classically used include: phosphotyrosine phosphatase inhibitors ammonium molybdate and sodium orthovanadate; acid phosphatase inhibitor sodium fluoride (NaF); secreted phosphatase inhibitor sodium tartrate; alkaline phosphatase inhibitor levamisole and phosphoserine/threonine phosphatases inhibitors okadaic acid and microcystin-LR [11–13]. Several biological roles for ectophosphatases have been proposed. These enzymes may provide microorganisms with a source of inorganic phosphate by hydrolyzing phosphomonoester metabolites [13–15] protect them upon entering the macrophage by suppressing the respiratory burst [16], as well as play a role in cell differentiation [17], infection of host cells [18–20] and protecting the cells from acidic conditions by buffering the periplasmic space with phosphate released from polyphosphates [21]. Some protein phosphatases have been described as being active towards low molecular weight nonproteic phosphoesters, such as alkyl and aryl phosphates, including the phosphotyrosine analog, *p-*nitrophenylphosphate [18]. From a general standpoint, the surface accessibility of ectophosphatases, along with protein phosphorylated on serine/threonine/tyrosine residues at the cell surface make this set of enzymes a key tool for the survival of pathogens in hostile environments and escaping the host immune responses [19, 22–24]. In this review, we describe the role of ectophosphatase activities in host-parasite interactions, particularly ectophosphatases in parasitic protozoa and fungi.

## 2. Ectophosphatase Activities in Protozoa Infection

Little is still known about the physiological role of protein phosphatase activity in trypanosomatids, even though the first demonstration of this activity in *Trypanosoma brucei* and *T. cruzi* took place in 1972 [25]. The kinetoplastid parasites have complex life cycles and some of their life forms are difficult to grow in culture, which may represent a problem for studying ectophosphatases. Pathogenic trypanosomatids have at least two different host environments in their life cycles, an insect vector and a mammal. Also, each trypanosomatid genus has different abilities to survive and reproduce in such hosts. For instance, *Leishmania *spp. are intracellular parasites, seeking to invade macrophages. On the other hand, *T. cruzi *invades and replicates in many cell-types, including macrophages, fibroblasts and myocytes. *T. brucei *is an exclusively extracellular parasite that resides in the bloodstream of the mammalian host. As the life cycles of these parasites take place through widely different environments, frequent and substantial adaptive changes are required in many cell processes, resulting in changes in gene expression, protein levels and protein modifications [26, 27]. Along with those, cell surface components play a key role in the survival of protozoan parasites in hostile environments and in confrontation with host immune responses. Since, these flagellates have an unusual composition of phosphatases with the PTP family being greatly reduced while the STP family is expanded by comparison with human phosphatases. The low similarity to their vertebrate counterparts indicates that these enzymes may be potentially suitable targets for development of potent inhibitors with minimal effects on the physiology of mammalian hosts [5]. 

Under these conditions, ectophosphatases play an important role in the interaction of cells with their surroundings, especially because their catalytic sites face the extracellular milieu. Ecto-phosphatases has been reported in some protozoa parasites, such as *T. rhodesiense *[28], *T. congolense *[29], *T. brucei *[30, 31], *T. cruzi *[32], *T. rangeli* [13, 33], some *Leishmania *species [11, 34], *Herptomonas muscarum muscarum* [35], *Phytomonas* spp. [36, 37], *Entamoeba histolytica* [38], *Giardia lamblia* [39] and *Trichomonas vaginalis* [40]. In general, these ectoenzymes are usually reported to have optimum activities in the acidic pH range, and they are therefore also known as membrane-bound acid phosphatases [28, 29]. In trypanosomatids, the low optimum pH and the surface location of these enzymes suggest its role in an acidic microenvironment and/or a close relationship with lysosomal digestion, possibly reflecting an adaptation of the parasite to the intracellular or phagosomal environment [41, 42]. 

Cloning and purification of an acidic phosphatase in *T. brucei* suggest that these enzymes may represent a new ectophosphatase class lacking homology to other known phosphatases [31]. It seems that these proteins are related to the regulation of *T. brucei* development, since these acidic phosphatases are expressed in bloodstream forms, but not in the insect procyclic form [31]. Likewise, an ectophosphatase activity on the surface of intact procyclic and bloodstream forms of *T. brucei* was demonstrated by Fernandes et al. [43, 44]. These enzymes show different behavior, like sensitivity to inhibitors and metal interference. Similarly, an ectophosphatase was also cloned and purified in *L. mexicana*, where it was located in the endosomal/lysosomal compartment between the flagellar pocket and the nucleus in wild-type promastigotes, and the overexpression of this protein leads to its abundant exposure on the cell surface [45, 46]. The same was seen with membrane-bound acid phosphatase from the bloodstream form of *T. brucei*, where the enzyme is supposed to participate in the maintenance of endocytosis/exocytosis and in differentiation to the insect stage [47]. The wide distribution of acid phosphatases on the cell may reflect some physiological adaptation for parasite survival within the host.

In this scenario, ectophosphatase activities were identified at the cell surface of all *T. cruzi* development stages: epimastigote [48], trypomastigote [18] and amastigote forms [18, 32]. It seems that in amastigote forms these enzymes are magnesium-dependent and can hydrolyse phosphoaminoacids and phosphoproteins under physiological conditions [18, 32]. This behavior could facilitate the interaction between parasite and host cells, once *T. cruzi* phosphatases leads to dephosphorylation of proteins important in the signal transduction pathway or cycle regulation of this protozoan parasite. Supporting this idea, Y strain presents Mg^2+^-dependent ectophosphatase activity, while Colombiana strain expresses Mg^2+^-independent activity [48]. Among other characteristics, members of these two groups have different patterns of behavior considering their ability to infect mammalian host cells. Parasites from the Colombiana strain appeared to be more infective to myoblasts than those from the Y strain, while the latter is more infective towards macrophages than the parasites of the Colombiana strain [49]. Intriguingly, platelet-activating factor (PAF), a phospholipid mediator involved in differentiation cellular in *T. cruzi*, induces the secretion of an ectophosphatase in these parasites, associating this event with the infectivity of the parasite [50]. 

Addition of sodium orthovanadate (a protein tyrosine phosphatase inhibitor) in the interaction medium from *L. amazonensis* and macrophages significantly increased parasite binding and internalization, suggesting that *Leishmania *induces tyrosine phosphorylation [24, 51]. Under these conditions, protein tyrosine kinase-linked pathways regulate the *Leishmania* promastigote invasion, which ectophosphatase activity upregulate *L. amazonensis *binding ligands for macrophage receptors and intracellular survival within these cells [24, 51, 52]. It seems that during macrophage infection by *Leishmania *the parasite attenuates MAP kinase signaling, as well as c-FOS and iNOS expression in macrophages, stimulating the phosphotyrosine phosphatase activity in these cells [53–55]. These findings suggest a mechanism for macrophage deactivation used by *Leishmania* spp. and possibly by other intracellular pathogens as a strategy of the parasites to interact and survive within their hosts. In *L. donovani* tyrosine phosphatase activity was also detected, suggesting that tyrosine phosphorylation occurs, though not via receptor tyrosine kinase or tyrosine kinase-like activities but very likely due to the activity of atypical and/or dual specific kinases [56]. Futhermore, a membrane-bound PTP has been describe in *L. major* metacyclic promastigote forms, which is translocated to the cytoplasm in promastigotes. In spite of the increased level of the molecule in metacyclic promastigotes compared to the procyclic forms, the specific activity of the enzyme was lower in metacyclic than in procyclic promastigotes [42]. Interestingly, a protein tyrosine phosphatase, has been identified in *L. major *(*Lm*PTP1) that allows amastigotes forms to survive in mice [57]. Although its biological function is unclear, this may be an important factor in virulence, enabling the invading pathogen to survive in a host. Ecto-phosphatase isolated from *L. donovani* promastigotes inhibits the production of superoxide anions in intact human neutrophils [16]. This activity could contribute to the survival of the parasite within the host, we can hypothesize that parasites with greater ectophosphatase activity would be more resistant to oxidative bursts from the host's immune system.

The role of ectophosphatases in invasive amoebiasis is still unknown, even though two acid phosphatases have been characterized in these parasites: a membrane-bound acid phosphatase (MAP) [58, 59] and a phosphatase that is secreted to the culture medium (SAP), as well as to the cell interface in amoebic liver abscess [60, 61]. These enzymes may be associated with cellular adhesion processes, since the invasive *E. histolytica* showed much higher ectophosphatase activity when compared to the noninvasive counterpart and the free-living *E. moshkovskii* [62].

## 3. Ectophosphatase Activities in Fungi Infection

The fungal cell wall is a compact albeit dynamic structure that plays important roles in several biological processes determining cell shape, morphogenesis, reproduction, cell-cell and cell-matrix interactions, osmotic and physical protection. Several different cell wall components have been characterized such as specific enzymatic activities, heat-shock proteins, glycosphingolipids (GSL), melanin, histone and integrin-like proteins [63]. These components have been exhaustively studied as putative targets for drug and immunotherapy. 

Even though the roles of ectophosphatases in fungi are still largely unknown, the cellular distribution of ectophosphatases, together with their ability to interfere with physiologic processes through the removal of phosphate groups of regulatory proteins, suggest a task for these molecules during the infection of host cells. The presence of surface-located acid phosphatases, called ecto or extracytoplasmic phosphatases has been demonstrated in nonpathogenic yeast *Saccharomyces cerevisiae* [64] and in pathogenic species such as *Candida albicans* [20], *Candida parapsilosis* [19, 65], *Sporothrix schenckii* [66], *Aspergillus fumigatus* [67], *Fonsecaea pedrosoi* [22, 68], *Cryptococcus neoformans* [23] and *Pseudallescheria boydii *[69].

Futhermore, most of the phosphatases synthesized under Pi-limiting conditions are either located on the extracellular medium or are associated with the plasma membrane or cell wall [15, 22]. Corroborating with this hypothesis, Kneipp et al. [22] demonstrated that conidial forms of *F. pedrosoi* has an ectophosphatase activity modulated by exogenous phosphate. It seems that in *F. pedrosoi,* conidial cells that were cultivated in a Pi-depleted medium had an ectophosphatase activity significantly higher than that of fungal cells grown in the complete medium. These cells expressing high phosphatase activity were significantly more capable of adhering to epithelial cells and fibroblasts than fungi expressing basal levels of enzyme activity [22]. It was then proposed that the removal of phosphate groups from surface proteins in host cells could result in conformational transitions and in an attenuated electrostatic repulsion between fungal and epithelial cells. Probably, the removal of inorganic phosphate could therefore expose at the host surface additional sites for interaction with infectious agents. It seems that ectophosphatases may contain adhesive domains that could directly promote the attachment of fungal cells to their hosts, therefore functioning similarly to the well-characterized microbial adhesins. Probably, they could regulate the functional activation of surface adhesins, which would be the key structures mediating fungal attachment. Intriguingly, known activators of signaling pathways and cell differentiation, PAF and propanolol, promoted an enhancement of *F. pedrosoi *ectophosphatase activity [17] suggesting that *F. pedrosoi *ectophosphatase may be considered a surface marker for morphological transition and infection.

In the fungus* C. neoformans* a thick capsule composed of neutral and charged polysaccharides [70], can be modulated by different environmental conditions, including the sites of fungal infection inside the host. It seems that the molecules coating the outer layer of the cell wall could be relevant during the interaction of poorly encapsulated cells with host tissues. Ectoenzymes possibly have their accessibility to external receptors masked by the capsule polysaccharides of *C. neoformans*, diminishing the potential of these structures to be surface molecules influencing the interaction between fungal and host cells. In fact, different isolates of *C. neoformans* express ectophosphatase activity [23]. However, the levels of enzyme activity, varied considerably among the isolates and no correlation between enzyme activity and capsular size or serotype was observed. Evidences show that isolates with capsular polysaccharides of the same serotype varied greatly in ectophosphatase activity. In addition, the strain, which is poorly encapsulated, removed phosphate groups much more efficiently than strain, which expresses a large capsule, indicating that the presence of the capsule impairs enzyme activity in this process. On the other hand, some encapsulated strains presented levels of ectophosphatase activity higher than that observed in the acapsular mutant. Moreover, some strains that had very similar levels of enzyme activity, but differ greatly in capsule size were also found [23]. Taken together, these data indicate that differences observed in enzyme activity should be derived from natural variation of ectophosphatase expression in different *C. noeformans* strains. Corroborating with the previous findings, Kiffer-Moreira et al. [19] investigated three different isolates of *C. parapsilosis*, including a laboratory-adapted strain (CCT 3834) and two recently isolated strains (RFO and H297). They observed that the RFO strain exhibits the highest levels of enzyme activity and adhesion to CHO cells, followed by the H297 and the CCT 3834 isolates. Pretreatment of yeasts with the irreversible inhibitor sodium orthovanadate caused a significant reduction in the ability of these fungi to attach to epithelial cells [19]. Although sodium orthovanadate can affect different biological processes [71] and inhibit ATPases involved in cation transport [72, 73], its major biological activity in living cells occur on the cell surface, as the oxidation–reduction reactions that take place in the cytoplasm diminish its inhibitory effect. *C.parapsilosis* ectophosphatase may be considered an important virulence factor. Similarly, *C. albicans* isolate from oral cavities of HIV-infected children (HIV^+^) present an ectophosphatase activity significantly higher than the HIV-negative children (HIV^−^) [20]. The *C. albicans* yeasts from HIV^+^ patients showed higher indices of adhesion to epithelial cells, which suggests that the activity of fungal acidic surface phosphatases may contribute to the early mechanisms required for disease establishment [20]. It is reasonable the hypothesis that ectophosphatases represent a virulence marker, since these enzymes represent part of the outer layer and are linked to cell differentiation and host cell-pathogen interactions.

## 4. Concluding Remarks

The balance of phosphorylation-dephosphorylation of serine, threonine and tyrosine residues modulates signaling pathways critical for determining the outcome of multiple cellular functions [74]. Ecto-phosphatases are enzymes able to hydrolyze phosphorylated substrates in the extracellular medium. Further studies are warranted to resolve the roles of ectophosphatases in host-pathogen interactions, as well as the possible correlations between the expression of these enzymes and the clinical manifestation of the diseases.
